# Immunomodulation by glycosylation: bridging glycobiology to adaptive immunity and therapeutic innovation

**DOI:** 10.3389/fimmu.2026.1729274

**Published:** 2026-03-20

**Authors:** Shengwen Ding, Han Wang, Zhaoxin Han, Senlian Hong

**Affiliations:** State Key Laboratory of Natural and Biomimetic Drugs, Chemical Biology Center, Department of Chemical Biology, School of Pharmaceutical Sciences, Peking University, Beijing, China

**Keywords:** adaptive immunity, glycoengineering, glyco-immune checkpoint, glycosylation, vaccination

## Abstract

Adaptive immunity provides specific and long-lasting protection through antigen-specific responses and immune memory, a property that has been successfully harnessed in transformative immunotherapies. Advancing these treatments requires a deeper understanding of the molecular mechanisms that fine-tune immune activity. In this context, glycosylation, a ubiquitous and complex post-translational modification, is emerging as a central regulator. By shaping the structure and function of immune proteins, glycans critically regulate the behavior of T cells, B cells and antigen-presenting cells. This review summarizes current knowledge on how glycosylation governs key processes in acquired immunity, from antigen recognition and immune signal transduction to cell-cell interaction. We highlight that glycans can function directly as antigens or, more commonly, by forming distinct glyco-signatures on cell surfaces that sculpt immune response. Furthermore, we discuss the therapeutic implications of these insights and outline emerging strategies that target glycosylation pathways to enhance anti-tumor immunity, improve vaccine efficacy, and treat autoimmune disorders. A deeper integration of glycobiology into adaptive immunity is therefore pivotal for developing the next generation precision immunotherapies.

## Introduction

Human cells are enveloped by a dense, complex layer of glycans known as the “glycocalyx”, which can extend up to 30 nm from the cell surface (1). The structural diversity of glycans arises from the linkage of monosaccharides via α- or β-glycosidic bonds, forming linear or branched chains (2–5). This complexity is further manifested by the covalent attachment of glycans to proteins, lipids, and RNAs (6–9). Over 50% of cellular proteins are glycosylated, profoundly influencing protein folding, stability and function (7). Together recent discovery of glycoRNA and its key role in multiple processes, these findings underscore the importance of glycans (10).

Adaptive immunity, renowned by antigen specificity and memory, is a cornerstone of host defense against pathogens and central pillar of modern immunotherapies. Given the pervasiveness of glycosylation, glycans are critical modulators of adaptive immunity (11). However, current research often remains siloed, focusing on glycans of specific targets. An integrated perspective that bridges glycobiology and immunology is therefore needed to fully appreciate glycosylation as a higher-order regulatory system. Here, we detailed recent advances to establish a conceptual framework in which glycosylation emerges as a master regulator of adaptive immunity and a promising frontier for therapeutic innovation. Specifically, the molecular mechanisms by which glycans modulate key adaptive immune processes were summarized. We then examine how aberrant glycosylation, particularly within the tumor microenvironment, contributes to immune evasion. Finally, we discuss the therapeutic implications of these insights and summarize emerging strategies that target glycosylation pathways for immune checkpoint modulation, metabolic intervention, and the development of next-generation vaccines.

## An overview of protein glycosylation

Protein glycosylation is categorized into several forms, classified according to glycosidic linkages. This section provides an overview of the major types of protein glycosylation and their biosynthetic pathways, laying the groundwork for understanding how these modifications regulate adaptive immune functions.

In 1955, Neuberger et al. identified the linking of carbohydrates to the amide group of the asparagine residue in ovalbumin via *N*-glycosidic bonds (12). *N*-glycosylation is a highly conserved process and is essential for the proper function of immunologically critical receptors and secreted factors (13). *N*-glycosylation initiates in the endoplasmic reticulum (ER) with the assembly of a lipid-linked precursor. Then, oligosac-charyltransferase (OST) complex transfer precursor to asparagine residue in motif (Asn-X-Ser/Thr, X≠Pro) (14–17). The properly folded glycoprotein is recognized by cargo receptors and packaged into coat protein complex II (COPII) vesicles for transport to the Golgi apparatus (18). Then the *N*-glycans are “remodeled” by glycosidases and glycosyltransferases, producing three major classes: high-mannose, hybrid, and complex *N*-glycans (18, 19).

*O*-glycosylation is defined by monosaccharide attached to the hydroxyl groups of serine or threonine residues (20). In Golgi apparatus, *N*-acetylgalactosamine (Gal*N*Ac) is added by polypeptide Gal*N*Ac-transferases (21, 22). Unlike classical glycosylation, *O*-Glc*N*Ac, discovered by Torres and Hart, involves a single *N*-acetylglucosamine modification to numerous nuclear and cytoplasmic proteins. *O*-Glc*N*Ac is added by *O*-Glc*N*Ac transferase (OGT) and removed by *O*-Glc*N*Acase (OGA), making it a highly dynamic sensor for nutrient status (23–26). Beyond *N*- and *O*-linked glycans, specialized forms of glycosylation fulfill critical, niche functions. Glycosylphosphatidylinositol (GPI) anchors are complex glycolipids attached to *C*-terminus of proteins in the ER, tethering them to the outer leaflet of the plasma membrane (27). This modification is crucial for localization and function of several immunoregulatory proteins, including CD14 and CD55. *C*-glycosylation is a relatively rare modification involving the covalent attachment of α-mannose residue to the indole ring of tryptophan via a C-C bond, mainly present in glycolipids, oligosaccharides, glycoproteins, and natural products (28).

## The adaptive immunity: specificity and memory

While innate immunity provides a rapid, first-line defense, adaptive immunity confers the hallmarks of antigen-specific recognition and immune memory for long-term protection (29, 30). Lymphocytes are the cornerstones of immune system, primarily T and B cells (31, 32). The efficacy of this system is shaped by post-translational modifications, among which glycosylation has emerged as master regulator. This section outlines key players and processes of adaptive immunity, establishing a framework for the immunological role of glycan.

T cells are classified into two major subsets: CD4^+^ helper T cells and CD8^+^ cytotoxic T cells (31). Both subsets develop in the thymus and express T-cell receptors (TCRs) that recognize antigens. Upon activation, CD4^+^ T cells proliferate and differentiate into distinct effector lineages (i.e., Th1, Th2, and Th17) with unique cytokine secretion profiles. CD8^+^ cells, in contrast, are the principal effectors of antiviral and anti-tumor immunity, directly recognizing and eliminating infected or malignant cells following activation. B cell recognize native, unprocessed antigens via the B cell receptor (BCR). Antigen engagement drives B cell activation and differentiation into antibody-secreting plasma cells. Antibodies are glycoproteins that neutralize pathogens, activate the complement cascade and promote opsonization, thereby facilitating engulfment and destruction of pathogens (32). In addition to antibody production, B cells function as professional APCs, internalizing antigen and presenting processed peptides to helper T cells. Beyond immune cells, adaptive immunity relies on a network of soluble signaling molecules. Cytokines regulate immune cell activation, proliferation, differentiation, and effector function, orchestrating both local and systemic immune responses (33). Chemokines act as chemo-attractants, guiding the migration of immune cells to sites of infection, inflammation, or lymphoid tissue organization. Together, these coordinated cellular and molecular interactions underpin the specificity and memory that define adaptive immunity.

## Glycosylation as a master regulator of adaptive immunity

The components including TCRs, BCRs, antibodies, cytokines and their cognate receptors are all glycoproteins. The glycans are not merely decorations; rather, they are modulators of protein conformation, stability, and intermolecular interactions. The cell- and protein-specific glycan repertoire, often referred to as glyco-code, dictates immune cell activation thresholds, directs cellular migration, and fine-tunes the balance between immune tolerance and effector responses. As such, glycosylation represents a central regulatory axis of adaptive immunity and an emerging focal point for therapeutic intervention.

### Tuning adaptive immunity through glycosylation

**Figure 1:**
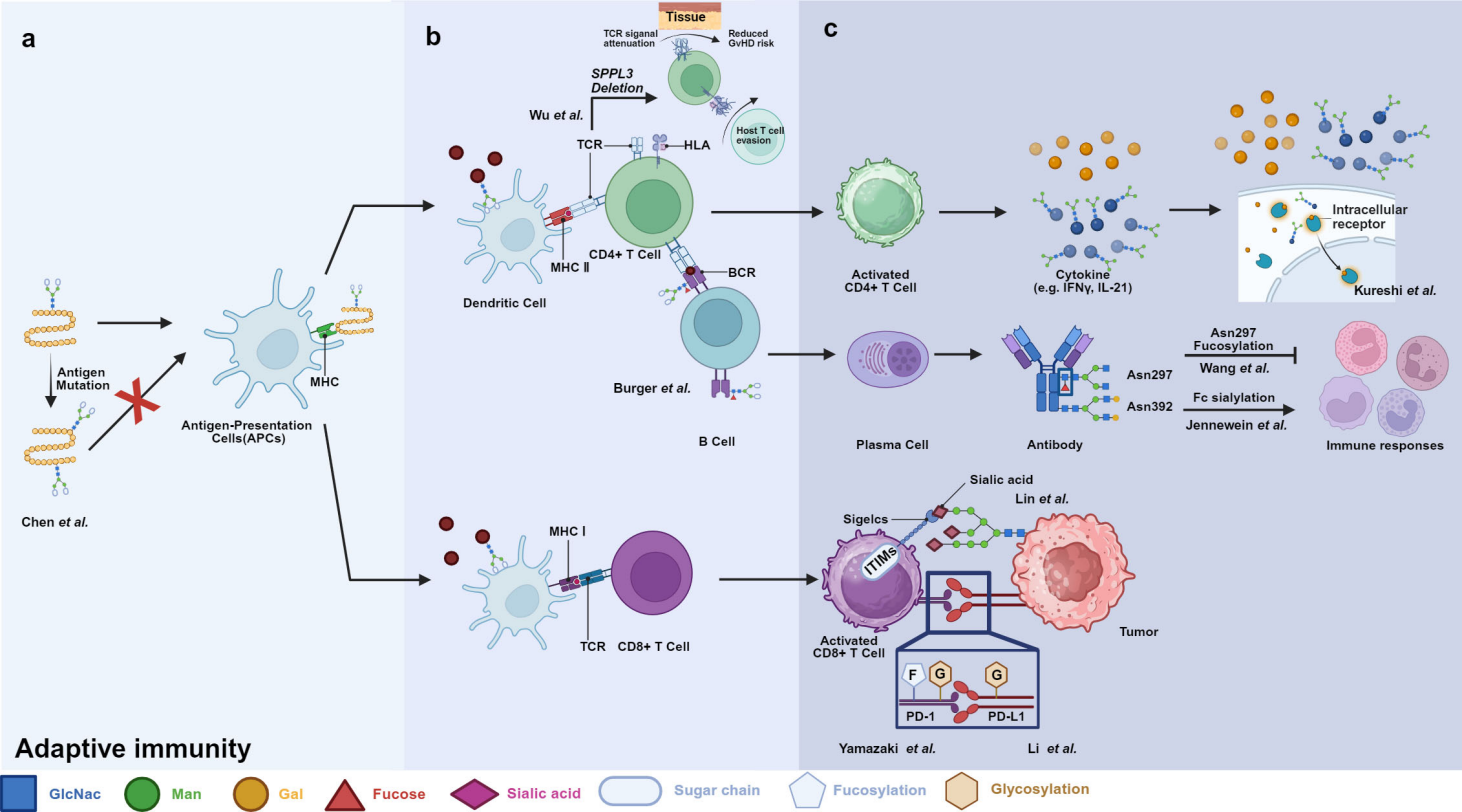
Schematic overview of glycosylation-dependent regulation in adaptive immunity. Glycosylation tightly regulates multiple stages of the adaptive immune response. **(a)** During antigen processing and presentation, antigen glycosylation influences recognition and processing by APCs. **(b)** Activation of T and B cells via TCR and BCR signaling is modulated by glycosylation of receptors and their ligands. **(c)** Glycosylation directly controls effector functions, including cytokine signaling, IgG induced immune responses and CD8^+^ T cell cytotoxicity. By serving as a dynamic regulatory layer at these critical events, glycans ultimately determine the balance between immune activation and tolerance.

Germline-encoded pattern recognition receptors (PRRs) traditionally sense conserved microbial structures. While antigen processing and presentation by antigen-presenting cells (APCs) typically initiate adaptive immune responses, effector immune cells can also directly engage glycan ligands through PRRs and lectin receptors, thereby shaping downstream adaptive response(**Figure 1a**). Aberrant glycosylation now recognized as a hallmark of immune dysfunction. A prominent example is cancer, where tumor cells frequently upregulate sialylated glycans (sialosides) on glycoproteins and glycolipids (11). Siaosides act as high-affinity ligands for sialic acid-binding immunoglobulin-like lectins (Siglecs) expressed on immune cells (34). Most Siglecs contain intracellular immune receptor tyrosine-based inhibitory motifs (ITIMs) or ITIM-like domains that, upon phosphorylation, recruit SHP phosphatases and suppress immune activation (35–37). Increased surface fucosylation is another prevalent glycan alteration in tumor. Fucosylated glycans can be recognized by C-type lectin receptors (CLRs), a large family of calcium-dependent glycan-binding proteins expressed on APCs (38, 39). Depending on the specific CLR engaged and its associated signaling motifs, these interactions can induce either immune activation or tolerance. In melanoma models, tumor-derived fucosylated antigens promote immune evasion by selectively engaging tolerogenic CLRs, thereby skewing immune responses toward suppression (40, 41).

Many carcinomas show a shift towards truncated, immature *O*-glycans, such as Tn (Gal*N*Acα1-*O*-Ser/Thr) and sialyl-Tn antigens (STn) (42). These structures are recognized by macrophage galactose lectin (MGL), a CLR expressed on macrophages and conventional dendritic cell subset 2 (cDC2), which modulates activation signaling (43, 44). Furthermore, sialylated *O*-glycan can inhibit immune activation via Siglecs engagement (44, 45). Beyond biochemical signaling, glycocalyx can physically mask antigens from recognition and simultaneously deliver inhibitory signals to immune cells.

### Antigen glycosylation determinates immunogenicity

Antigen recognition by adaptive immune receptors is a prerequisite for immune activation. BCRs recognize native antigens based on three-dimensional structure, whereas TCRs recognize peptide antigens presented by major histocompatibility complex (MHC) molecules on APCs (46). In the MHC-I pathway, endogenous antigens are degraded into peptides of 8–11 amino acids, transported into ER, loaded onto MHC-I, and presented to CD8^+^ T cells (46, 47). In contrast, MHC class II molecules present larger peptides (10–16 amino acids) derived from exogenous antigens, thereby activating CD4^+^ helper T cells (48).

Glycosylation critically influences antigen uptake, processing, and presentation. Certain structures serve as ligands for APCs, facilitating antigen presentation (**Figure 1b**). For example, Liu et al. engineered a glycan-decorated nanoparticle vaccine that enhanced dendritic cell uptake and antigen presentation (49). Glycans also directly shape antigenicity and immunogenicity. A Thr160Lys mutation in viral hemagglutinin (HA) introduces a novel N-glycosylation site, enabling immune evasion by shielding epitopes from pre-existing antibodies (50, 51). Similarly, altered glycosylation in cancer can impair antigen processing and presentation, thereby promoting immune escape. Glycoconjugate vaccine exploit glycan immunogenicity by coupling glycans to carrier protein (52). Following APC uptake, the carrier protein is processed into peptides presented on MHC II, driving T-cell activation and enabling robust B-cell responses, affinity maturation, and immune memory. Wang et al. developed a Globo-H-based cancer vaccine that undergoes intracellular glycan processing within DCs, generating smaller glycans presented via MHC II (53).

### T cell glycosylation regulates adaptive immunity

Immune checkpoint pathways are tightly regulated by glycosylation. Programmed cell death protein 1 (PD-1) contains core fucosylation at N49 and N74 mediated by fucosyltransferase 8 (FUT8). Inhibition of core fucosylation reduces PD-1 surface expression, enhances T-cell activation, and augments antitumor immunity (54, 55). PD-L1, the principal ligand for PD-1, is heavily glycosylated, and glycosylation protects it from proteasomal degradation (56). Costimulatory receptors are similarly regulated. CTLA-4 suppresses T cell responses by competing with CD28 for binding to B7 ligands (CD80/CD86) on APCs (57, 58). CD28 is extensively N-glycosylated, with glycans accounting for nearly half its molecular mass (59). Disruption of CD28 *N*-glycosylation, either by mutagenesis or glycosylation inhibitors, increases its affinity for CD80 and amplifies downstream signaling, demonstrating that *N*-glycans can negatively regulate CD28 function (57, 60). Metabolic pathways further intersect with glycosylation. Reduced mannose metabolism contributes to T-cell dysfunction (61), while glucose-fueled glycosphingolipid biosynthesis is essential for CD8^+^ T-cell expansion and cytotoxicity (62).

### Glycosylation governs NK cell functions

NK cells are innate lymphoid cells that eliminate virally infected and malignant cells without prior sensitization. Approximately 2 × 10¹^0^NK cells exist in the human body, constituting ~1% of immune cells and ~2% of lymphocytes (63, 64). NK cells are classified into NK1 (CD56^dim^CD16^+^), NK2 (CD56^bright^CD16^−^) and NK3 (CD16^dim^ adaptive NKG2C^high^). NK cell activity is regulated by a balance of activating and inhibitory receptors, many of which are glycosylated. N-glycosylation sites on NKp30 (e.g., N42, N69, N121) are essential for receptor oligomerization and cytotoxic function (65). Core fucosylation is indispensable for NK-cell biology; FUT8 deficiency impairs CD122 expression, leading to defective NK-cell survival, maturation, and antitumor immunity (66).

### B-cell glycosylation regulates adaptive immunity

Activated B cells differentiate into plasma cells that secrete high-affinity antibodies and act as cytokine producers, releasing both pro-inflammatory (e.g., TNF-α, IFN-γ) and anti-inflammatory (e.g., IL-10, TGF-β) mediators (62). BCR signaling is initiated by antigen binding to membrane-bound immunoglobulin coupled to the CD79a/CD79b heterodimer, which mediates intracellular signal transduction (67, 68). Dysregulated BCR glycosylation is a defining feature of B-cell malignancies. In chronic lymphocytic leukemia (CLL), aberrant glycosylation of CD79a and CD79b leads to protein misfolding, reduced surface expression, and impaired BCR signaling (69, 70). In follicular lymphoma, somatic hypermutation can introduce novel N-glycosylation sites into immunoglobulin variable regions, altering antigen recognition and promoting malignant transformation (71).

### Glycosylation regulates complement immune responses

The complement system composes over 50 soluble and membrane-bound proteins, serves as a critical interface between innate and adaptive immunity. Glycans act as pathogen recognition tags, antibody molecular switches, and quality-control elements for complement proteins (72, 73). Complement activation enhances adaptive immunity by augmenting B-cell responses, shaping T-cell differentiation, and linking antibody production to effector mechanisms (72). This system is activated via three primary pathways, including the classical, lectin-based, and alternative pathway (74). The lectin pathway is initiated by carbohydrate recognition on microbial surfaces, triggering antibody-independent complement activation (75, 76). In the classical pathway, IgG galactosylation promotes Fc hexamerization and high-avidity C1q binding (77). Complement component C3, a central convergence point of all activation pathways, carries two high-mannose *N*-glycans essential for its function (78). Alterated C3 glycosylation has been linked to autoimmune pathology, including type I diabetes, where immature glycoforms correlate with loss of immune tolerance (79). These findings underscore that complement activity is intrinsically governed by glycan signatures.

### Glycosylation regulates IgG functions

Immunoglobulins are the most abundant glycoproteins in serum, with IgG being the predominant isotype. IgG structure, stability, half-life, and Fc-mediated effector functions are tightly governed by glycosylation (**Figure 1c**). Loss of IgG glycosylation abolishes immune function and leads to severe immune dysregulation (80, 81). Each IgG Fc domain contains a conserved *N*-glycosylation site at Asn297 within the CH2 domain. These glycans maintain Fc conformation, and even subtle compositional changes can induce major structural and functional shifts (82). Core fucose removal enhances binding to FcγRIIIa on NK cells, dramatically increasing antibody-dependent cellular cytotoxicity (ADCC) (83, 84), whereas fucosylation suppresses this interaction (85). This principle underlies the development of afucosylated therapeutic antibodies. Fc sialylation confers anti-inflammatory properties by promoting interactions with lectin receptors and FcγRIIb, leading to the induction of regulatory cytokines (86, 87). Galactosylation enhances complement activation by increasing C1q binding and modulates inflammatory responses, as observed in COVID-19 severity and other inflammatory diseases (88, 89). While Fc glycosylation is highly conserved, approximately 15–20% of IgG molecules also carry *N*-glycans within the Fab region, where they modulate antigen binding, pharmacokinetics, and half-life. IgG glycosylation patterns also serve as dynamic biomarkers of health and disease (90, 91). In Crohn’s disease, increased agalactosylated IgG2 glycoforms precede clinical onset and promote pro-inflammatory innate immune activation (92).

## Glycan-centric approach to regulate adaptive immunity

The ability to precisely edit glycans has created a paradigm shift in immunotherapy. Because glycosylation controls immune recognition, receptor signaling, trafficking, and checkpoint function, glyco-engineered immune cells and glyco-targeted biologics are increasingly positioned as key components of next-generation therapeutic platforms (**Table 1**). Below, we summarize three major translational directions: (i) glyco-engineering of immune cells, (ii) targeting tumor-associated glycans to disrupt immune evasion, and (iii) metabolic interventions that reshape glycosylation and immune function.

**Table 1:** Summary of clinically glycan-centric therapies, checkpoints and current clinical trial phase.

Glycan-centric modulation of adaptive immunity			
Methods	Cell type	Disease	Clinical trial
Glyco-engineering of immune cells for enhanced cell therapies	T cell	B-cell non-Hodgkin lymphomas (95)	Phase I
	γδ T cell	Ovarian cancer (95)	Preclinical studies
	Dendritic cell	Tumor vaccine (95)	Preclinical studies
	NK cells	B-Cell Lymphoma (95)	Preclinical studies
	NK cells	B-cell acute lympho-blastic leukemia (95)	Phase 0
Methods	Molecular targets	Disease	Clinical trial
Targeting tumor-associated glycans to disrupt immune evasion	Siglec-2(CD22) 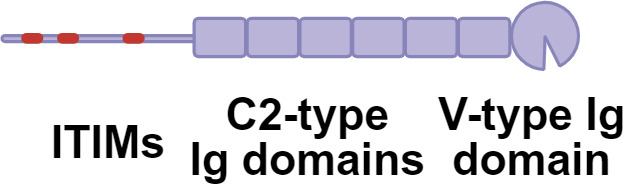	B-Cell Lymphoma (100)	Phase I
	Siglec-3(CD33) 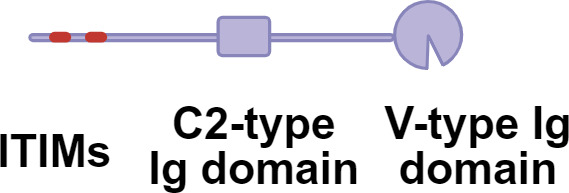	Acute myeloid leukemia (100)	Phase I
	Siglec-7(CD33rSiglec) 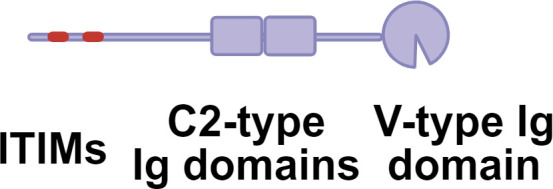	Acute myeloid leukemia (100)	Preclinical studies
	Siglec-15 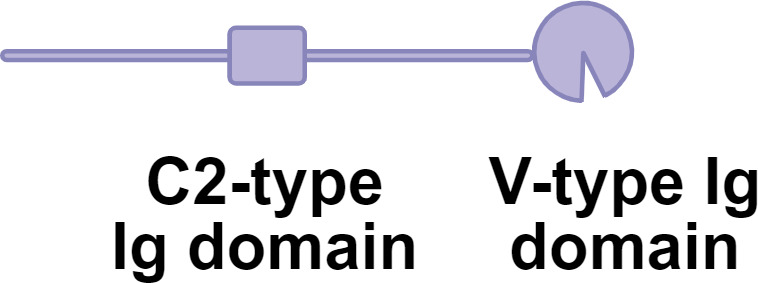	Non-small cell lung cancer (100)	Phase II
	PD-1	Enhance antitumor immunity (100)	Preclinical studies
	MUC1-Tn 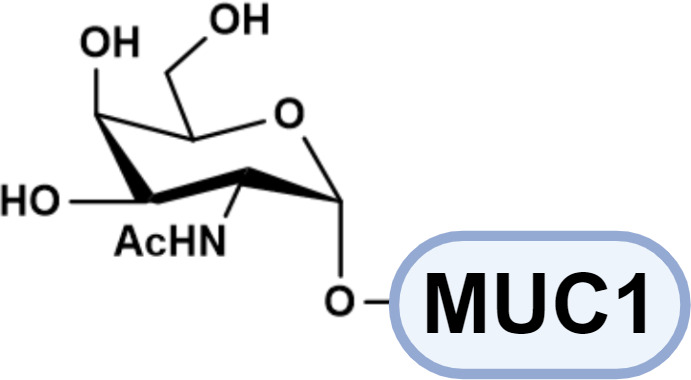	Breast cancer (100)	Preclinical
		MUC1-Tn-positive intrahepatic cholangiocarcinoma (100)	Preclinical
	Sialylated Tn (STn) 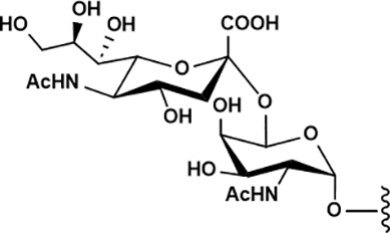	Ovarian cancer (104, 105)	Phase I
	GD2 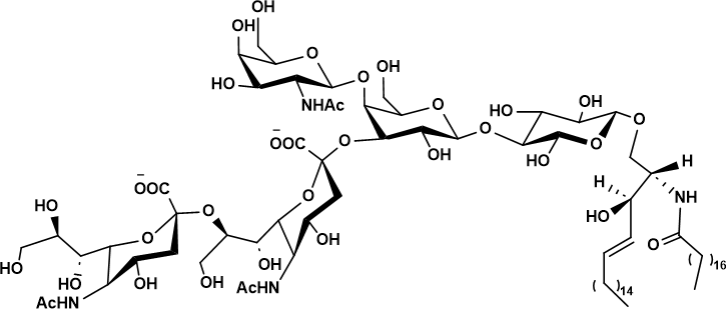	Neuroblastoma (104, 105)	Phase I
		Diffuse midline gliomas (105)	Phase 0

### Glyco-engineering of immune cells for enhanced cell therapies

Glyco-engineering can improve the efficacy of cell-based therapies by modulating immune cell persistence, tumor targeting, and functional activation states (93, 97). One major limitation of allogeneic CAR-T therapy is host immune rejection. To address this, Wu et al. developed an innovative strategy by knocking out SPPL3, a negative regulator of glycosylation. SPPL3 deletion increase global surface *N*-glycosylation, which reduces immune recognition and protects CAR-T cells from FasL-mediated apoptosis. Importantly, this “glycan shield” mitigated allogeneic rejection and reduced graft-versus-host disease (GvHD) risk while preserving cytotoxic function, supporting the development of more effective “off-the-shelf” CAR-T products (93). Beyond αβ T cells, γδ T cells are promising for immunotherapy but often exhibit limited tumor-targeting efficiency. Chen et al. introduced a modular chemical strategy by anchoring antibodies onto γδ T cells through metabolic incorporation of unnatural sialic acids bearing a bioorthogonal handle, enabling efficient and versatile surface engineering for improved therapeutic performance (98). Similarly, DC vaccines can be potentiated by glycan editing. Zhuang et al. constructed a glycopolymer-engineered DC vaccine via metabolic glycoengineering and copper-free click chemistry, achieving enhanced antitumor efficacy (99). NK cells provide potent innate cytotoxicity but typically lack precise tumor-homing specificity. Hong et al. used a chemoenzymatic approach to install synthetic glycan ligands on NK-92MI and primary NK cells, equipping them with high-affinity ligands for CD22, a receptor highly expressed in B-cell malignancies. This glycan editing redirected NK cells toward CD22-positive tumor targets and significantly enhanced binding and killing *in vitro* without harming healthy cells (97). Extending this concept, Jin et al. engineered primary NK cells by metabolically incorporating a sialic acid derivative, promoting CD22 ligand display and improving targeting of CD22^+^ B-cell malignancies. A phase 1 NK-cell trial in B-cell acute lymphoblastic leukemia further underscores translational potential (100).

### Targeting tumor-associated glycans to disrupt immune evasion

Many cancers exhibit elevated surface sialylation, enabling engagement of inhibitory Siglecs and promoting immune suppression. As a result, Siglecs and tumor-associated sialoglycans are increasingly viewed as a class of glyco-immune checkpoints (11, 44, 45). Therapeutically, reducing tumor sialylation can attenuate inhibitory signaling and enhance immune-mediated tumor killing. Multiple approaches are under development, including sialic acid mimetics and anti-Siglec antibodies, with growing evidence of synergy when combined with conventional checkpoint inhibitors (11, 94). Glycosylation-dependent regulation also extends to canonical checkpoint pathways. Because PD-1 and PD-L1 function and stability are influenced by glycosylation, glyco-epitope-targeting therapeutics represent a rational next step. For example, STM418 is a monoclonal antibody designed to recognize glycosylated PD-1, particularly at the N58 glycan site, thereby blocking PD-1/PD-L1 interactions with reportedly improved inhibitory potency compared with conventional anti–PD-1 antibodies and enhanced antitumor activity (101).

Tumor-associated glycan epitopes also offer direct targets for engineered cell therapies. MUC1-Tn is broadly overexpressed in malignancies (42); Zhai et al. engineered MUC1-Tn- targeting CAR-T cells and demonstrated antitumor activity in breast cancer mouse models, and subsequent studies support efficacy in MUC1-Tn-positive intrahepatic cholangiocarcinoma (ICC) (102, 103). The STn antigen is likewise enriched in malignant cells (95), and dual-targeting CAR-T designs have shown improved antitumor activity compared with STn-only CAR-T strategies (96). In parallel, early clinical exploration of CAR-T targeting glycan-associated antigens such as TAG-72 has been reported, including trials in metastatic colorectal cancer with implications for other solid tumors (e.g., ovarian cancer) (104). Another extensively explored glyco-antigen is GD2 (disialoganglioside 2), overexpressed in neuroblastoma, melanoma, sarcoma, and glioblastoma (105). GD2-targeting CAR-T therapies have been developed for solid tumors including high-grade glioma, osteosarcoma, and neuroblastoma, with several programs advancing through phase I evaluation (106, 107). However, major challenges remain, including on-target/off-tumor toxicity, limited infiltration into solid tumors, and variable efficacy. As glyco-targeted CAR-T products expand, regulatory pathways for CAR-T have continued to evolve; nevertheless, the distinctive nature of glycan epitopes (including heterogeneity, tissue distribution, and antigen dynamics) suggests that more tailored regulatory frameworks and evaluation standards may ultimately be needed for glyco-engineered cellular therapeutics.

### Metabolic intervention as immunotherapy

Beyond targeting specific glycoproteins, an alternative strategy is to modulate monosaccharide availability and metabolism, thereby reshaping cellular glycosylation and immune cell function. Tumors exhibit high glucose demand (Warburg effect), motivating dietary interventions such as low-carbohydrate ketogenic approaches intended to reduce glucose availability and support anticancer therapy through metabolic stress and immune activation (108, 109). However, systemic glucose modulation can produce unintended immunological consequences. Wu et al. reported that dietary glucose restriction or impaired local glucose metabolism can suppress primary tumors but paradoxically promote lung metastasis (110). Fructose provides a second example of metabolic complexity. While fructose can fuel tumor growth, its effects are context dependent and can extend to multiple cellular compartments. In Apc-/- models colorectal cancer models, long-term moderate fructose intake increased tumor incidence through activation of glycolysis and fatty acid synthesis pathways (111). Feng et al. further showed that combined fructose and glucose exposure increased the NAD^+^/NADH ratio via sorbitol dehydrogenase activation, enhancing metastatic potential (112). Fructose can also shape stromal remodeling: Cui et al. found fructose uptake by colorectal cancer cells promoted proliferation and activation of cancer-associated fibroblasts (CAFs), generating a pro-metastatic niche through tumor–stroma metabolic coupling (113). Conversely, Fang et al. reported that limiting fructose intake suppresses angiogenesis and tumor progression (114). Importantly, fructose restriction strategies must be approached cautiously, as fructose may also support immune competence. Zhang et al. showed that dietary fructose can modulate adipocyte metabolism to enhance antitumor CD8^+^ T-cell responses and control tumor growth (115). Collectively, these studies highlight that metabolic modulation is a double-edged strategy: while it may constrain tumor growth, it can also reshape immune surveillance and tissue-specific immunity in unpredictable ways. More systematic preclinical evaluation and well-controlled clinical studies are therefore required to define when and how metabolic interventions can be safely leveraged for glyco-immunotherapy.

## Glycans as targets and tools for vaccination

Vaccination is among the most successful public health interventions, leading to the eradication or effective control of diseases such as smallpox and polio (116). The fundamental principle of vaccination is to safely mimic infection, thereby priming adaptive immunity to generate durable, antigen-specific immune memory. Central to vaccine design is the identification of stable, highly immunogenic antigens that can be efficiently captured, processed, and presented by APCs. Glycans represent a particularly powerful but complex class of vaccine antigens. Allergies arise from overreaction of immune system to harmless substances, and glycans are frequently the causes. For example, peanut allergy is driven primarily by Arah1, which serves as IgE-binding epitopes. abolishes its allergenicity, directly demonstrating the immunological potency of glycan structures (117). This strong immunogenicity reflects the profound structural differences between host and pathogen glycans. Pathogens often express non-human monosaccharides, unusual glycosidic linkages, and distinct three-dimensional patterns that are absent in mammalian systems, making pathogen-associated glycans ideal vaccine target.

### Antibacterial glycoconjugate vaccines

**Figure 2:**
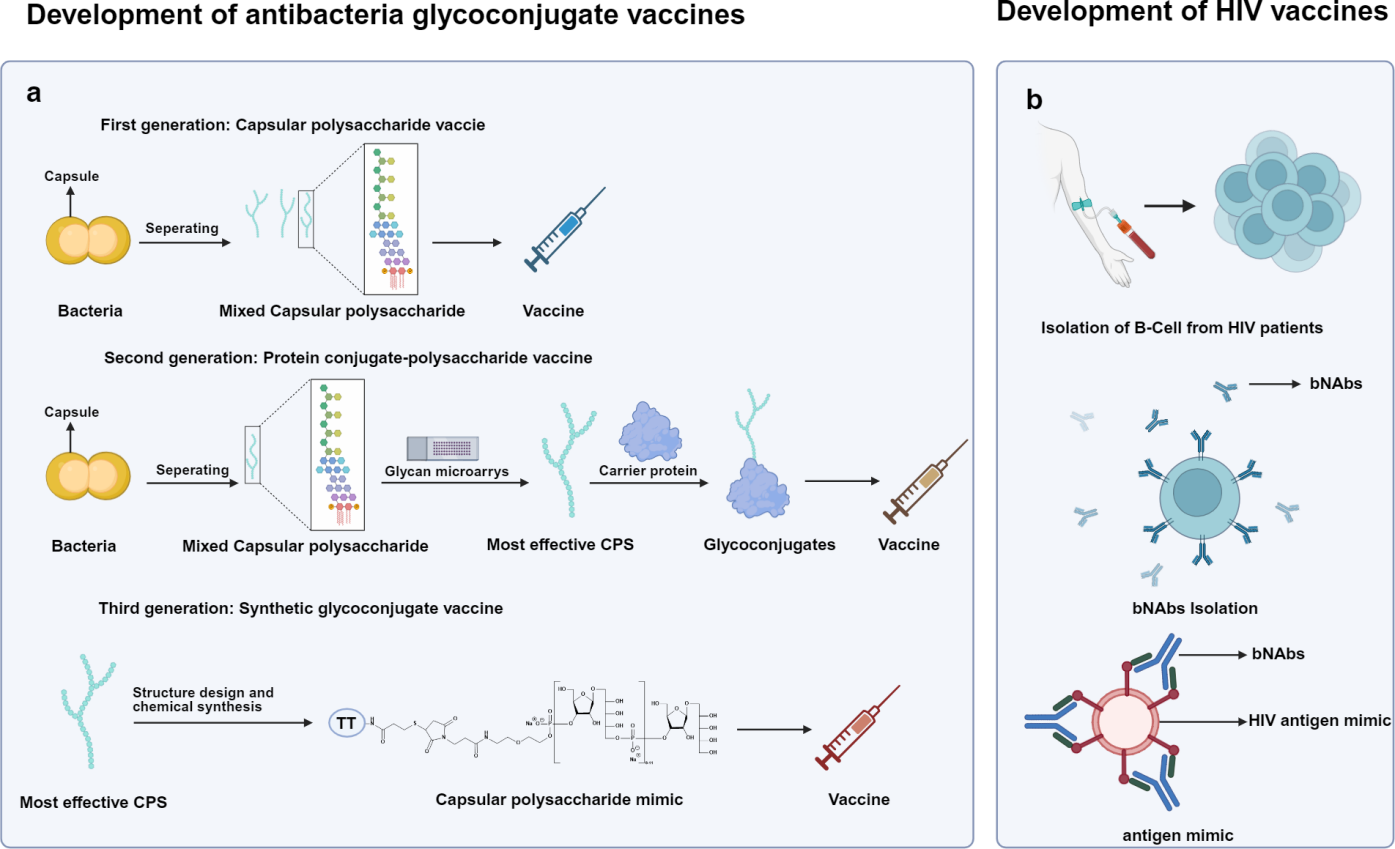
Paradigm shifts in glycan-based vaccine design. Glycan-based vaccines have evolved through distinct technological paradigms, including antibacterial glycoconjugates and HIV-1 immunogens. **(a)** Development of antibacteria glycoconjugate vaccines: First-generation vaccines used isolated capsular polysaccharides (CPS) directly. Second-generation vaccines employed glycan microarray screening to identify effective CPS epitopes, which were conjugated to carrier proteins to boost immunogenicity. Third-generation vaccines employed structure design and chemical synthesis to produce precise CPS mimics. **(b)** Development of HIV vaccines: HIV vaccines development is relies on a bNAb-based approach: B cells isolated from HIV patients generate bNAbs, which are then used to identify and optimize HIV antigen mimics capable of inducing protective antibody responses.

The surfaces of many pathogenic bacteria are coated with dense layers of capsular polysaccharides (CPS), which are chemically distinct from human glycans (118, 119). The development of polysaccharide-based bacterial vaccines has progressed through three major generations (**Figure 2a**).

Capsular polysaccharide vaccine (first-generation):In the early 20th century, Dochez et al. demonstrated that serotype specificity of *Streptococcus pneumoniae* is determined by its CPS (120). Vaccines composed of purified bacterial polysaccharides, such as those against *pneumococcus*, *meningococcus*, *Haemophilus influenzae type b* (Hib), and *Salmonella typhi*, induce T-cell- independent immune response (121). While effective in adults, these vaccines suffer from major limitations: poor immunogenicity in infants and young children, induction of low-affinity antibodies (primarily IgM), and failure to generate immune memory (122, 123).

Carrier−polysaccharide vaccine (second-generation): To overcome these limitations, bacterial CPS were chemically conjugated to immunogenic carrier proteins, converting immune response from T-cell-independent to T-cell-dependent. In this strategy, B cells recognize the polysaccharide component, while APCs internalize and process the carrier protein, presenting peptide fragments to CD4^+^ T helper cells. This interaction drives class switching, affinity maturation, and the generation of long-lived memory B cells (124). Conjugate vaccines represent a major milestone in vaccinology and remain among the most successful antibacterial vaccines to date.

Synthetic glycoconjugate vaccines (third-generation): More recently, antigen production has shifted from biological extraction to chemical synthesis. Quimi-Hib, the first and currently only licensed synthetic glycoconjugate vaccine, consists of chemically synthesized oligosaccharides that mimic the CPS of Hib and are conjugated to a carrier protein (52). These synthetic antigens, based on defined repeats of ribosylribitol phosphate, eliminate batch-to-batch variability and contamination risks. Notably, studies have shown that a tetrameric form of the synthetic oligosaccharide conjugate elicits the most effective immune response (125). To date, tens of millions of doses of Quimi-Hib have been produced worldwide, validating synthetic glycoconjugates as a robust platform for future vaccine development.

Despite success, glycoconjugate vaccines face several challenges. Glycosidic bonds can be hydrolyzed by the abundant glycosidases, threatening antigen stability before effective immune priming occurs. Efficient induction of long-lasting T cell–dependent immunity also relies on optimal antigen processing and presentation, but glycan-only antigens are generally less efficiently presented by MHC molecules. Moreover, certain glycan structures may elicit undesirable immune responses, including allergic reactions. Achieving maximal immunogenicity while minimizing adverse effects remains a central challenge. Compared with protein antigens, the recognition, processing, and presentation of glycoconjugate vaccines remain incompletely understood, and standardized methods to evaluate these processes are still lacking. In addition, the chemical synthesis, purification, and reproducible conjugation of complex glycans remain technically demanding.

### The glycan shield of human immunodeficiency virus: a target and a barrier

HIV poses one of the most formidable challenges in vaccine development. Its envelope glycoprotein (Env), composed of gp120/gp41 heterodimers assembled into a trimer, is densely shielded by glycans that mask underlying protein epitopes from immune recognition (126, 127). Nevertheless, a subset of infected individuals eventually develop broadly neutralizing antibodies (bNAbs) capable of recognizing glycan-dependent epitopes (128, 129) (**Figure 2b**).

Early vaccine strategies aimed to recapitulate epitopes recognized by known bNAbs (128). One of the first identified bNAbs, 2G12, binds clusters of high-mannose glycans on gp120 (130, 131). Li et al. engineered synthetic scaffolds displaying multiple Man_9_GlcNAc_2_ glycans to mimic the 2G12 epitope (132). Through iterative optimization, including fluorination and galactosylation, binding affinity to 2G12 was substantially improved (133, 134). To enhance immunogenicity, these glycan antigens were presented multivalently on platforms such as bacteriophage Qβ virus-like particles (VLPs) (135). Although these vaccines elicited glycan-specific antibodies, the induced responses preferentially targeted accessible mannose residues rather than the native glycan architecture on the gp120 trimer, resulting in a lack of neutralizing activity (136, 137). This outcome underscored the challenge of immunodominance and epitope mismatch.

Subsequent efforts shifted toward more complex bNAb epitopes, such as those recognized by the PGT and PG antibody families, which neutralize over 70% of global HIV strains (127, 131). These antibodies recognize composite epitopes consisting of conserved peptide motifs in the V3 loop and specific high-mannose glycan sites. Wang et al. chemically synthesized V3 loop glycopeptides bearing native-like glycans (e.g., Man_9_GlcNAc_2_ at N332), conjugated them to T-helper epitopes and adjuvants, and demonstrated broad cross-reactivity with gp120 proteins (138, 139). However, these vaccines still failed to elicit antibodies capable of neutralizing live HIV-1, highlighting the difficulty of recapitulating the precise structural and conformational requirements. The natural development of bNAbs in patients is a prolonged, multiyear process involving extensive somatic hypermutation (140). Interestingly, children can develop potent and broadly neutralizing responses much more rapidly than adults (141), as exemplified by BG505, who generated antibodies capable of neutralizing 91% of Tier 2 viruses (142, 143). These observations inspired germline-targeting vaccine strategies aimed at selectively activating bnAb precursor B cells. Nelson et al. f demonstrated that multidose immunization with lineage-designed SOSIP trimers could initiate early bnAb-lineage responses (144). In a phase I clinical trial, the immunogen GT1.1 administered with the AS01B adjuvant, a liposome adjuvant system composed of 3-O-desacy-4-monophoryl lipid A(MPL) and a triterpene saponin(QS-21), showed excellent safety and successfully activated VRC01-class bnAb precursor B cells in most recipients. Antibodies isolated from vaccinated individuals displayed somatic hypermutation and neutralized sensitive-tier HIV-1 pseudoviruses (145).

Despite these advances, major challenges remain. The extreme heterogeneity of gp120 glycans, combined with conformational masking and rapid viral mutation, complicates the design of universally effective immunogens. Innovative strategies are being explored to overcome these barriers, including engineering Env trimers with selective glycan deletions near conserved sites such as the CD4-binding site. Fabian-Alexander et al. near-native trimers with targeted *N*-glycan deletions that recruited appropriate B cell responses, yielding antibodies capable of neutralizing nearly 70% of a global HIV-1 panel (146). Wang et al. further demonstrated that combining vaccination targeting the fusion peptide with simian–human immunodeficiency virus infection elicited potent and broadly neutralizing plasma responses (147). In summary, while HIV vaccine development remains exceptionally challenging, continued advances in glycan engineering, structural vaccinology, and immunogen design suggest that glycan-focused strategies may ultimately enable effective and durable protection.

## Discussion

Adaptive immunity is the cornerstone of human immunity, and glycans are recognized as master regulators. Their broad immunomodulatory capacity underscores the central role of glycobiology in shaping next-generation immunotherapies. However, the full landscape of glycosylation in adaptive immunity remains incompletely mapped, in part due to limitations in analytical technologies capable of resolving dynamic, cell-specific glycan heterogeneity *in vivo*. Glycans function not only as direct antigens but also as critical modulators of antibody immunogenicity, immune receptor activity, and intercellular communication. Glycosylation of immune cells and immune effector molecules dynamically fine-tunes activation thresholds, effector functions, and tolerance. Glycan-based vaccines exemplify the translational success of glycobiology, having saved countless lives through effective prevention of infectious diseases. Against this backdrop, this review has examined therapeutic advances that span from early polysaccharide vaccines to synthetic glycoconjugates and the evolving frontier of HIV vaccine design.

A central theme emerging from these studies is the multifunctionality and context dependence of glycan-mediated regulation. While many current strategies target site-specific glycosylation changes, the pervasive nature of glycans means that therapeutic manipulation can produce broad and sometimes opposing biological effects. For example, inhibition of glucose metabolism may restrict tumor growth but paradoxically facilitate metastatic spread by reshaping immune and stromal niches. Similarly, targeting sialylation to enhance antitumor immunity may disrupt immune tolerance and increase the risk of systemic autoimmunity. These trade-offs highlight the necessity of adopting a system-level perspective when designing glycan-targeted interventions. Improved tools for glycan analysis, manipulation, and visualization are needed to define stage- and cell-type–specific glycan functions across immune responses. Also, the discovery of optimal glycan antigens, the chemical synthesis and stabilization of complex carbohydrate structures, and their precise delivery to immune cells continue to pose technical and conceptual obstacles. Ultimately, a deeper and more integrated understanding of glycobiology within adaptive immunity will be essential for transforming glycan-targeted concepts into safe, effective, and durable immunotherapies.
